# Targeting tissue immune memory in chronic-remitting autoimmune kidney diseases: current concepts and future therapies

**DOI:** 10.1093/ckj/sfag299

**Published:** 2026-09-07

**Authors:** Malte Hellmig, Christian F Krebs

**Affiliations:** III. Department of Medicine, University Medical Center Hamburg-Eppendorf, Hamburg, Germany; Hamburg Center for Translational Immunology (HCTI), University Medical Center Hamburg-Eppendorf, Hamburg, Germany; Hamburg Center for Kidney Health (HCKH), University Medical Center Hamburg-Eppendorf, Hamburg, Germany; III. Department of Medicine, University Medical Center Hamburg-Eppendorf, Hamburg, Germany; Hamburg Center for Translational Immunology (HCTI), University Medical Center Hamburg-Eppendorf, Hamburg, Germany; Hamburg Center for Kidney Health (HCKH), University Medical Center Hamburg-Eppendorf, Hamburg, Germany

**Keywords:** ANCA, crescentic glomerulonephritis, immune memory, lupus nephritis, T cell

## Abstract

Immune-mediated kidney diseases have a substantial disease burden and frequent disease relapses significantly contribute to the high morbidity. Relapsing disease courses are associated with poor outcome and accelerated progression to end-stage renal disease. The pathomechanisms leading to relapse are diverse and only incompletely understood. Both adaptive and innate immune cells are implicated in the immune memory of these diseases. In addition to antigen-dependent mechanisms of T and B cells, persistent epigenetic and metabolic changes in adaptive and innate immune cells may sustain immunological memory and thereby provide a cellular and molecular basis for relapse. Treatment options, especially in frequently relapsing cases, are limited and memory immune cells often evade standard immunosuppressive therapies. As our understanding of immunological memory continues to improve, new treatment options emerge. Improved depletion of B cells, antigen-specific cell-based therapies and redirecting epigenetic alterations may target long-lasting immune memory. This could reduce relapse risk and improve long-term outcomes in patients with immune-mediated kidney diseases.

## INTRODUCTION

Immune-mediated kidney diseases are a major cause of end-stage renal disease and have a high individual as well as socioeconomic disease burden [1, 2]. These diseases often present in a systemic form and are characterized by the recurrence of inflammation in the tissue. Immune-mediated kidney diseases are under the control of vastly different immunological mechanisms. Over recent years, therapeutic options have expanded and become more specific, driven in part by advances in immunotherapy and concepts adapted from oncology. However, robust clinical trial evidence in immune-mediated kidney diseases remains limited so far. Most immune-mediated kidney diseases have a chronic-remitting disease course; up until now, we have no reliable biomarkers available for the prediction of relapse and the resulting kidney damage.

Beyond initial immunological disease control, recurrent inflammation accelerates progression to end-stage renal disease and is associated with poor patient outcomes [3, 4]. Common among immune-mediated kidney diseases is the limited data on specifically managing these recurrences. Evidence for the specific management of disease recurrence is sparse, and therapeutic options are often limited or exhausted. Additionally, relapses of several immune-mediated kidney diseases can occur even after kidney transplantation, highlighting the need for a better understanding of the immunopathogenesis.

Pathomechanistically, it could be hypothesized that the immunological memory is a central mechanism driving disease recurrence. In this article we outline the most important molecular and cellular components of immunological memory and disease recurrence, providing kidney disease–specific mechanisms with the current evidence. In addition, we highlight potential future developments for specifically targeting the immune memory in immune-mediated kidney diseases.

## INNATE AND EPIGENETIC MEMORY

Recent data suggest that innate immune cells can exert functional memory-like states through epigenetic, metabolic or transcriptional reprogramming [5]. External or internal factors might modulate the innate immune cell population and induce long-term changes to their immunological responses. This phenomenon, commonly referred to as trained immunity, describes a memory-like functional state of innate immune cells. Such innate immunological memory was previously described in macrophages as well as in natural killer (NK) cells [6, 7] and at first only in response to infection or vaccination, for example protection against viral infections after Bacillus Calmette-Guérin vaccination [8]. Key epigenetic mechanisms underlying immune memory are DNA methylation and regulation of chromatin accessibility by histone acetylation or methylation [9, 10]. Exposure of innate immune cells to inflammatory stimuli can induce persistent changes in histone marks (Fig. 1). An increase of epigenetic modifications like H3K4me3 at promoters of inflammatory genes or H3K27ac at enhancer regions can lead to an increase of cytokine expression, like tumor necrosis factor-α, interleukin (IL)-6 and IL-1β [11].

**Figure 1:**
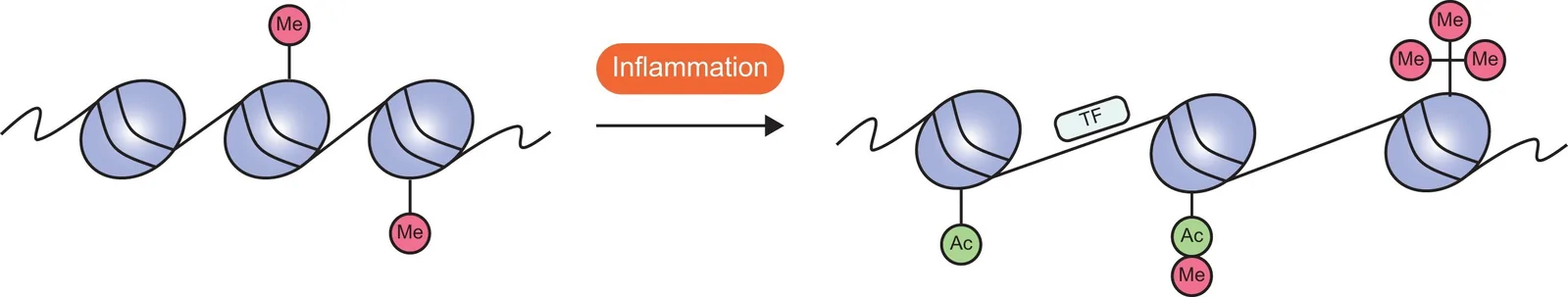
Mechanism of trained immunity. Inflammatory stimuli induce persistent epigenetic changes, including DNA methylation and histone modifications, which increase the accessibility of proinflammatory gene loci. These epigenetic modifications persist even after the inflammatory stimulus is resolved.Recurrence of inflammatory signals increases transcription factor binding and thereby the expression of proinflammatory genes.

However, while the mechanism of trained innate immunity is described in the context of infectious diseases, the evidence for kidney autoimmune diseases is limited, even though it can be assumed that this is a fundamental mechanism conserved in innate immune responses. In the context of systemic lupus erythematosus (SLE), different features of the innate and epigenetic modifications have been observed. For example, demethylation of interferon-related genes was identified in monocytes in patients with SLE leading to an increase of type I interferon signaling [12]. In addition, dysregulation of the innate immune response with loss of tolerance in neutrophils in antineutrophil cytoplasmic antibody (ANCA)-associated vasculitis has been described [13], but links to metabolic or epigenetic modifications as a result of inflammation are still missing.

Immune memory by epigenetic and metabolic modifications is not limited to the innate immune response, but also observed in adaptive immune cells. The pivotal first step in the pathomechanisic model of immunoglobulin A (IgA) nephropathy is the aberrant glycosylation of IgA, produced by activated B cells [14, 15]. B cells from patients with IgA nephropathy have been shown to exhibit an induction of DNA hypermethylation upon *in vitro* stimulation, associated with a notable increase in production of aberrant glycosylated IgA which might be mediated by a decrease in *COSMC* expression, a chaperone playing a relevant role in O-linked glycosylation of proteins [16, 17]. This mechanism not only supports the 4-hit model in IgA nephropathy [15] but also might be relevant in cases of disease relapse, as DNA methylations often persist for long times. However, persistent memory-like mechanisms independent of traditional adaptive immune memory responses are not only present in IgA nephropathy, but also observed in other immune-mediated kidney diseases like SLE. Here, preclinical models displayed metabolic reprogramming by inhibition of mitochondrial metabolism and glycolysis in CD4^+^ T cells that could revert inflammatory activity in lupus-prone mice [18].

Although epigenetic memory in the context of immune-mediated kidney diseases is not yet fully understood, some common maladaptive processes in epigenetic modifications are already identified and treatment options are developed. Apabetalone, an epigenetic therapy targeting BET proteins, is currently being evaluated in cardiovascular disease [19] and showed a potential reduction of major cardiovascular events in patients with chronic kidney disease and high cardiovascular risk [20]. This drug is a small molecule targeting bromodomain and extraterminal (BET) proteins, epigenetic reader proteins which bind histone acetylation and promote transcription factor assembly. BET inhibition reduces transcription of proinflammatory genes. Up until now, apabetalone has not been evaluated in the context of immune-mediated kidney disease, so it is unknown whether this drug will have beneficial effects. Besides BET inhibition there are some additional epigenetic modulators in clinical use in the field of oncology such as DNA methylation inhibitors (decitabine for acute myeloid leukemia and azacytidine for myelodysplastic syndrome), histone methylation inhibitors (tazemetostat in follicular lymphoma) or histone deacetylase inhibitors, such as vorinostat in T cell lymphoma, most of which have described adverse renal events [21]. Data supporting the use in immune-mediated diseases is missing.

Trained immunity might also play a role in kidney transplantation. In patients receiving kidney transplantation a study by Jonkman *et al*. could link suppression of trained immunity to an increase of long-term graft survival [22]. In a gene accessibility analysis from peripheral blood mononuclear cells after kidney transplantation, an increase of H3K4me3 and H3K27ac accessibility was observed and this could be linked to leukocyte activation. Additionally, persistent pro-inflammatory monocytes were detected at increased frequencies at 6 months after kidney transplant [23].

In a retrospective analysis, an association of delayed graft function with increased rate of acute rejection after 1 year was observed [24]. It could be hypothesized that the acute allograft injury leads to an innate memory response, which might promote allograft rejection. Mechanistically, dectin-1-expression macrophages could sense vimentin exposed by apoptotic cells, leading to epigenetic reprogramming of macrophages into an innate memory phenotype [10].

Through epigenetic and metabolic reprogramming, trained immunity can render innate immune cells hyperresponsive and may thereby contribute to disease recurrence. Notably these mechanisms are also present in adaptive immune cells. The development of trained immunity might be triggered by infection and response to foreign antigens, but also to microbiota, microbial metabolites or metabolic inflammation [25]. Regarding these probable causative factors, a better understanding is needed, especially in the context of relapsing immune-mediated diseases.

## B CELL MEMORY

Autoantibodies are among the main contributors for immune-mediated kidney diseases, although their contribution to disease pathogenesis can vary significantly. Diseases with a high relevance to their respective autoantibodies include THSD7A- and PLA2R-associated membranous nephropathy [26, 27] as well as anti-glomerular basement membrane (GBM) disease [28]. In these diseases binding of these antibodies to their specific target, PLA2R as a surface protein on podocytes or type IV collagen of the GBM, directly induces injury of the kidney [29, 30] and in case of PLA2R-associated membranous nephropathy also predicts outcome [31]. In the case of renal involvement in ANCA-associated vasculitis and SLE, autoantibodies are also involved in the immunopathogenesis, but the correlation with disease activity is limited. According to the lack of clinical correlation of ANCA titers with disease activity, in ANCA vasculitis guidelines do not recommend using ANCA titers alone for specific treatment decisions [32]. Although these diseases differ substantially in how autoantibodies mediate tissue injury, they all depend to some extent on B-cell responses and the generation of long-lived memory B cells.

In ANCA-associated glomerulonephritis (ANCA-GN) the anti-neutrophil cytoplasmic antibodies are a defining feature of the disease in many cases. Compared with other antibody-mediated immune diseases, the autoantibodies have not been shown to be pathogenic on their own. In the current understanding of the immunopathogenesis, autoantibodies, namely against myeloperoxidase or proteinase 3, prime neutrophils by binding to their surface antigens, which makes the neutrophils prone to activation and degranulation [33]. ANCA-GN is characterized by a frequently relapsing disease course [34–36]. To this end, B-cell targeting therapy with anti-CD20 monoclonal antibody rituximab has become the established standard for maintenance therapy in ANCA-GN [32] as well as a relevant part of induction therapy when combined with steroids. In some patients, renal vasculitis remains insufficiently controlled despite complete depletion of peripheral B cells with rituximab, resulting in relapse even during maintenance therapy. Moreover, relapse occurs in a substantial proportion of patients after completion of maintenance therapy. In some cases, not even initial immunological control is achieved despite usage of high-dose steroids in combination with rituximab plus cyclophosphamide, leading to refractory disease.

The cause of this recurrence might be memory B cells or plasma cells, which are located within bone marrow, lymphoid tissue or in the kidney [37, 38]. Due to their late differentiation state, CD20 expression is downregulated, allowing long-lived B cells and plasma cells escape rituximab treatment (Fig. 2). To target long-lived B cell memory, anti-CD38 therapy such as daratumumab has shown promising results in case reports of recurrent and refractory ANCA-GN [39], but it is not approved for the treatment of vasculitis, including ANCA-GN. Daratumumab had initial approval for treatment of multiple myeloma [40]. It specifically binds CD38, a protein expressed by plasma cells, eliminating these cells by complement-dependent cytotoxicity. Notably, CD38 is also expressed by other immune cells such as NK cells or T cells and this could explain additional therapeutic effects of daratumumab compared with rituximab. However, broader CD38 expression may also increase the risk of adverse events. Targeting plasma cells and other immune cells with anti-CD38 treatment might have beneficial effects in IgA nephropathy. A recent phase 2a study evaluated felzartamab in patients with IgA nephropathy and could show a sustained decrease in proteinuria and lower estimated glomerular filtration rate decrease after 2 years [41]. A phase 3 study is currently recruiting (NCT06935357).

**Figure 2:**
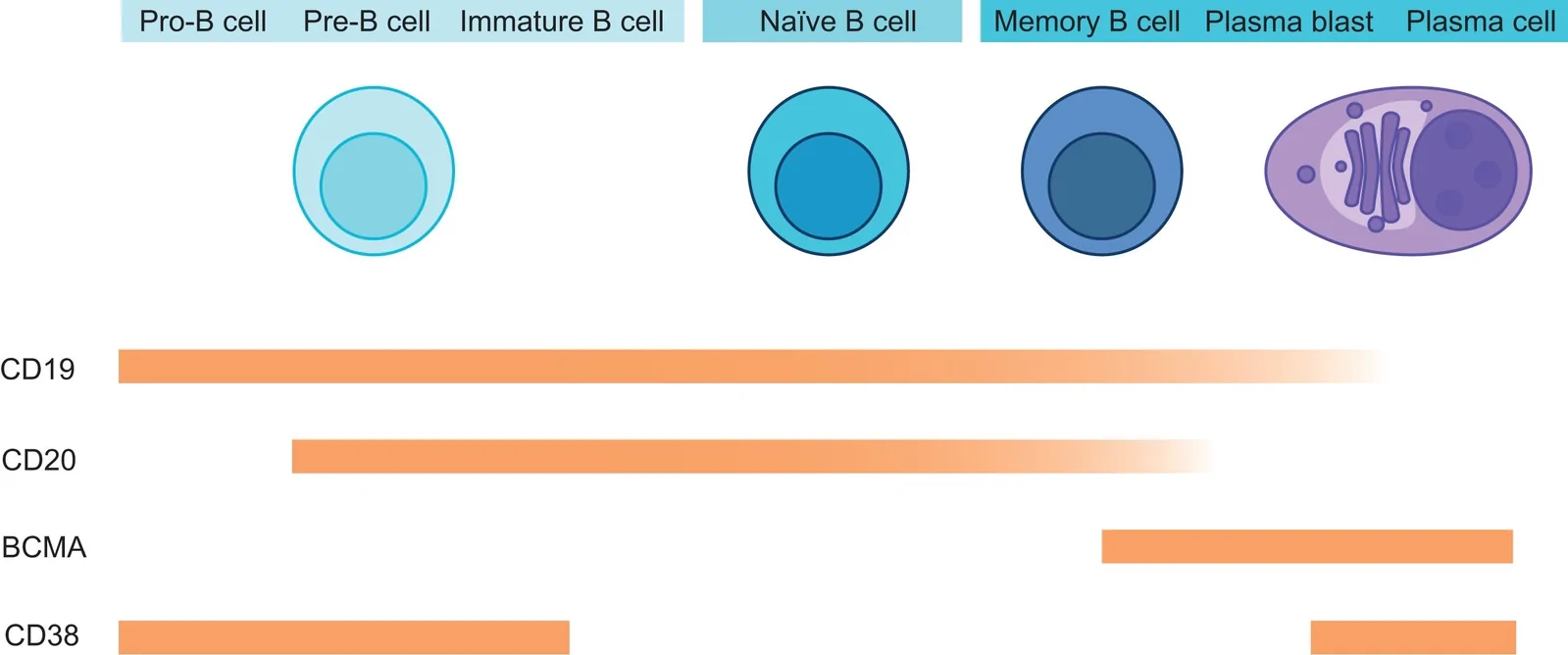
Expression of B cell surface markers during lineage development. CD19 and CD20 are broadly expressed in B cells. Notably, for both antigens expression is decreasing in late maturation of B cells, which enables the escape of pathogenic B cells and plasma cells from established B cell targeting therapies. To enable depletion of memory B cells and Plasma cells, BCMA and CD38 targeting therapy is required.

Besides escape of memory B cells from CD20-targeting therapy, persistence within the tissue provides a therapeutic challenge. Although rituximab efficiently depletes CD20^+^ B cells from the peripheral blood, tissue penetration may be insufficient, allowing pathogenic B cells to persist in sites such as the kidney. To overcome the tissue escape, new advances in cell-based immunotherapy provide a promising therapeutic approach. Chimeric antigen receptor (CAR)-T cells, first established as oncological therapy in lymphoma, show promising results especially as last-resort therapy in recurrent and refractory immune-mediated kidney diseases including ANCA-GN [42]. The CAR-T cells can penetrate the tissue deeply and deplete long-lived B cells using CD19- or BCMA-specific chimeric T cell receptors (Fig. 3). CAR-T cell therapies are currently evaluated in several multi-disease basket trials including many immune-mediated kidney diseases (NCT06775912, NCT06462144). However, CAR-T cell therapy is an invasive induction treatment associated with high long-term risks, significant costs, and considerable complexity. Therefore, these ongoing trials are indeed very important for a final assessment of this approach.

**Figure 3:**
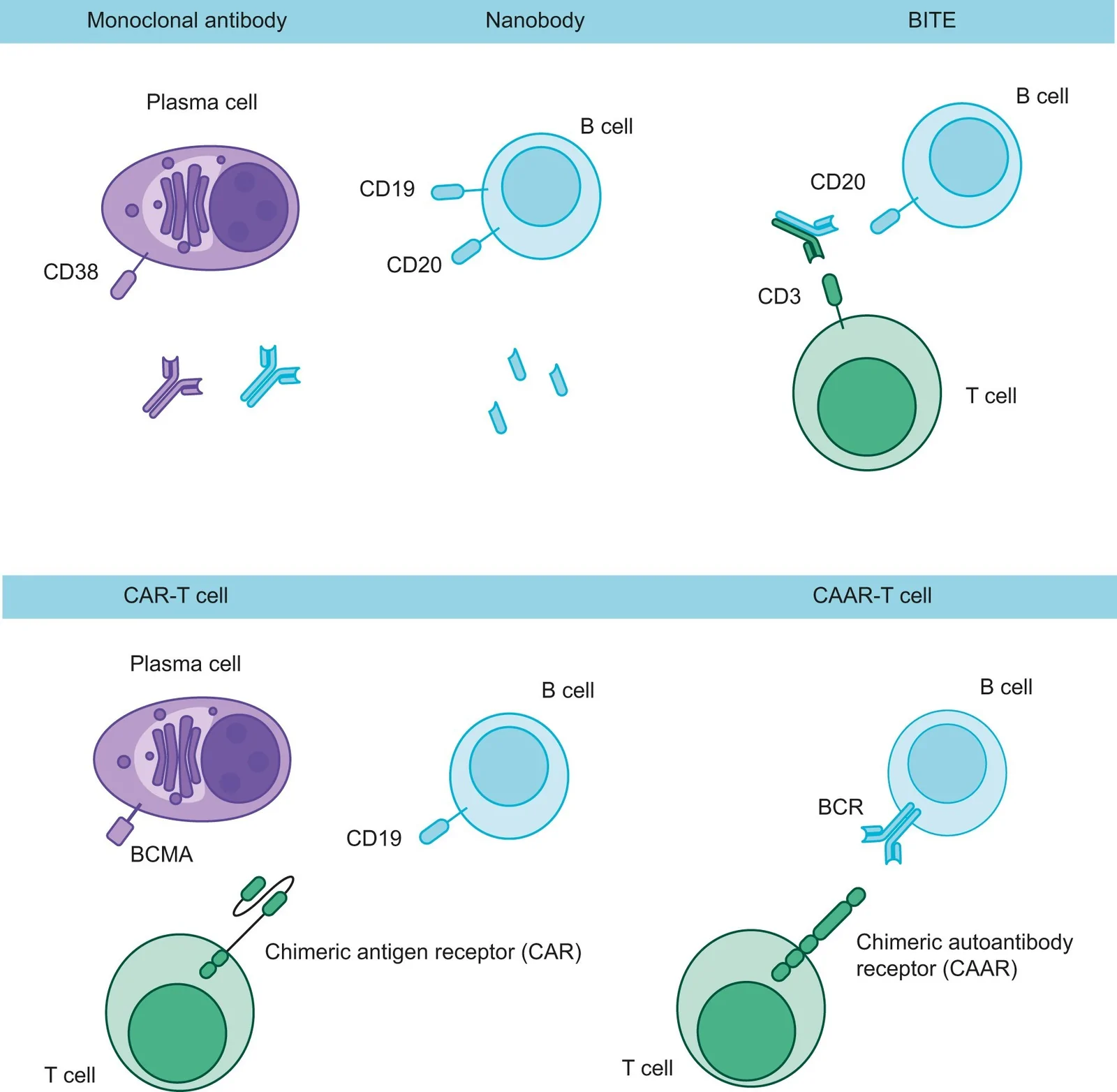
Concepts of B cell targeting therapies. Surface antigens on B and plasma cells, like CD38, CD19 and CD20 can be targeted by monoclonal antibodies or nanobodies. Bi-specific antibodies can improve antigen-specific depletion of B cells by directing T cell effector function. CAR-T cells can target B and Plasma cell specific surface antigens. CAAR-T cells target antigen specific B cells via an antigen domain that binds to the specific B cell receptor (BCR).

Lupus nephritis (LN) is the renal involvement of SLE, an autoimmune disease in which the pathomechanism significantly depends on B-cell activation and memory B cells. Like ANCA vasculitis, SLE also shows a significant degree of disease recurrence after initial remission [43, 44]. Pathomechanistically, autoreactive and hyperactive B cells produce anti-dsDNA antibodies which lead to formation of immune complexes and subsequent complement activation. Deposition of immune complexes in the kidney can induce endocapillary proliferation and podocyte injury, leading to a progressive kidney disease. Although autoreactive B cells play a central role in the LN pathogenesis, type I anti-CD20 targeting therapy with rituximab failed to prove efficacy in the LUNAR trial [45]. Interestingly, subsequent B cell–targeted approaches showed more favorable results, again suggesting that incomplete depletion of tissue-resident B cells may limit therapeutic efficacy. Most notably, the REGENCY trial investigating obinutuzumab, a type II anti-CD20 monoclonal antibody, showed improved outcomes compared with standard therapy [46]. Moreover, providing a higher level of antibody-dependent cellular cytotoxicity, obinutuzumab also shows a depletion of tissue B cells, most likely due to better tissue penetration [47], making obinutuzumab an interesting therapeutic option not only for LN. BAFF and APRIL are important cytokines for maturation and survival of B cells. In LN, the monoclonal anti-BAFF antibody belimumab is approved for the treatment of LN. However, the impact of BAFF on immune memory might be limited since long-lived plasma cell do not depend on this cytokine [48].

IgA nephropathy is an immune-mediated kidney disease in which B cells and plasma cells play a fundamental role. According to the current concept, B cells and plasma cells, originating from the gut, produce galactose-deficient IgA1, which contributes to immune complex formation and renal deposits and subsequent immune complex glomerulonephritis [15]. Besides B cell–memory mechanisms, a dysregulated mucosal immune tolerance seems to play an important role [49], enabling long term survival of B cells in the gut and contributing to disease activity [50]. Targeting the reservoir sites with topical budesonide and thereby suppressing B cell activation is a promising therapeutic option [51]. Although this concept is highly specific for IgA nephropathy, identifying such reservoir sites to better target autoreactive B cells and plasma cells might be a strategy applicable for other immune-mediated kidney disease. Recent studies introduce specific B cell–targeting therapies for the treatment of IgA nephropathy. Treatment with atacicept, a combined BAFF and APRIL inhibitor, could show a reduction of protein-to-creatinine ratio and serum Gd-IgA1 levels after 36 months [52]. Comparable results were reported in the interim analysis of a phase 3 trial evaluating sibeprenlimab treatment, a monoclonal antibody against APRIL [53].

In the case of IgA nephropathy, disease flares remain a therapeutic challenge even after kidney transplantation (Table 1), which might be a result of persistent dysregulation of memory B cells. After transplantation higher levels of serum IgA were associated with a higher rate of recurrent of the underlying IgA nephropathy [54]. A Japanese cohort of patients after kidney transplantation with a history of IgA nephropathy underwent tonsillectomy 1 year after transplantation and showed a lower rate of recurrences compared with the control group [55]. In the tonsillectomy group a reduction of serum levels of Gd-IgA1 and mesangial deposition of IgA were observed. It could be hypothesized that long-lived mucosal memory B cells, responsible for the production of Gd-IgA1, might be removed by tonsillectomy, although data B cells phenotype within the tonsil of patients with IgA nephropathy as well as validation of the intervention with cohorts in other countries are missing.

**Table 1:** Recurrence rates of immune-mediated kidney diseases.

Disease	Recurrence rate (follow-up time)	Recurrence rate after kidney transplantation (follow-up time^b^)
MPGN^a^		
IC-MPGN		
C3G		41% [86]
DDD		100% [87]
C3GN		57%–66% [87, 88]
IgA nephropathy	33% (6.3 years) [89]	13%–46% [90]
IgA vasculitis	15% (2 years) [91]	11.5% (10 years) [92]
ANCA glomerulonephritis	9.5–44% (5 years, 4 years) [34, 35]	17.3%–36.8% (not reported, 3.7 years) [93, 94]
Anti-GBM disease		<10% [86]
LN	32–55% (11.1 years, 3.6 years) [43, 44]	2.4–54% (not reported, 8 years^b^) [95, 96]
Membranous nephropathy	37% (5 years) [97]	48% [98]
PLA2R positive		60–83% [86]
Antibody negative		28–53% [86]
Primary FSGS		10–56% [86]

a Secondary causes are ruled out.

b Publications used different definitions of disease relapse, explaining the marked difference. C3G, complement 3 glomerulopathy; C3GN, complement 3 glomerulonephritis; DDD, dense deposit disease; FSGS, focal segmental glomerulosclerosis; IC-MPGN, immune complex-mediated membranoproliferative glomerulonephritis; MPGN, membranoproliferative glomerulonephritis.

In kidney transplantation, treatment of chronic active antibody-mediated rejection (cABMR) remains a major challenge regarding loss of graft function [56, 57]. For the cABMR a variety of targets have been evaluated, including B cell–targeting therapy. B cell–targeting therapy has been suggested. The evidence for rituximab in this context is very limited [58] and plasma cells targeting therapy with bortezomib showed a lack of efficacy [59]. However, targeting long-lived plasma cells with anti-CD38 antibodies, like daratumumab or felzartamab, showed promising results in smaller cohorts in reducing microvascular inflammation and donor-derived cell-free DNA [60, 61], but larger controlled studies are clearly needed to validate the results. The chimeric HLA antibody receptor T cells (CHAR T cells) are another promising therapeutic concept [62]. Here, engineered T cells specifically target HLA-antibody producing B cells and thereby might eliminate donor-specific antibodies. This treatment is currently in early preclinical stages and so far has been evaluated using *in vitro* assays and murine models.

Besides major improvement in depth of B cell–depleting therapy there are major advances in highly specific and autoantigen targeting therapy using chimeric autoantibody receptor (CAAR)-T cells [63]. This novel approach uses modified T cells expressing autoantigen domains fused to surface proteins. By binding the B-cell receptor of autoreactive B cells to this chimeric autoantigen domain, cell depletion is mediated (Fig. 3), representing a promising antigen-specific approach to pathogenic B-cell depletion in autoimmune diseases. Compared with previous therapeutic options, CAAR-T cells eliminate B cells regardless of their differentiation state, thereby also targeting long-lived memory and plasma cells which escape previous approaches. A phase 1 trial for desmoglein 3 chimeric autoantibody receptor T cells to treat pemphigus vulgaris is currently running. There are also interesting preclinical works for using CAAR-T cells for treatment of PLA2R-associated membranous nephropathy [64].

## T CELL MEMORY

Besides the B-cell memory response, T cells play an important role in many of the immune-mediated kidney diseases [65, 66]. T cells are critically involved in the early stages of the immune-mediated kidney diseases by recruiting innate and B cells. More importantly for relapse and disease persistence, recent studies have demonstrated the presence of tissue-resident memory T cells (Trm) in many autoimmune diseases and also in immune-mediated kidney diseases, including ANCA-GN [67] and LN [68]. The Trm cells can develop from both CD8^+^ as well as CD4^+^ cells and reside in tissue sites even after inflammation is cleared. These cells can persist long-term within tissues and may be reactivated in an antigen-independent manner [67]. Thereby, Trm cells may play a major role in mediating disease recurrence by leading to a self-reinforcing inflammatory cycle once activated (Fig. 4). Although the number of these cells is relatively low, their central role is the orchestration of inflammatory responses for example by attracting other immune cells. In contrast to the B cell–targeting therapy, specifically targeting pathogenic T cells is less well established.

**Figure 4:**
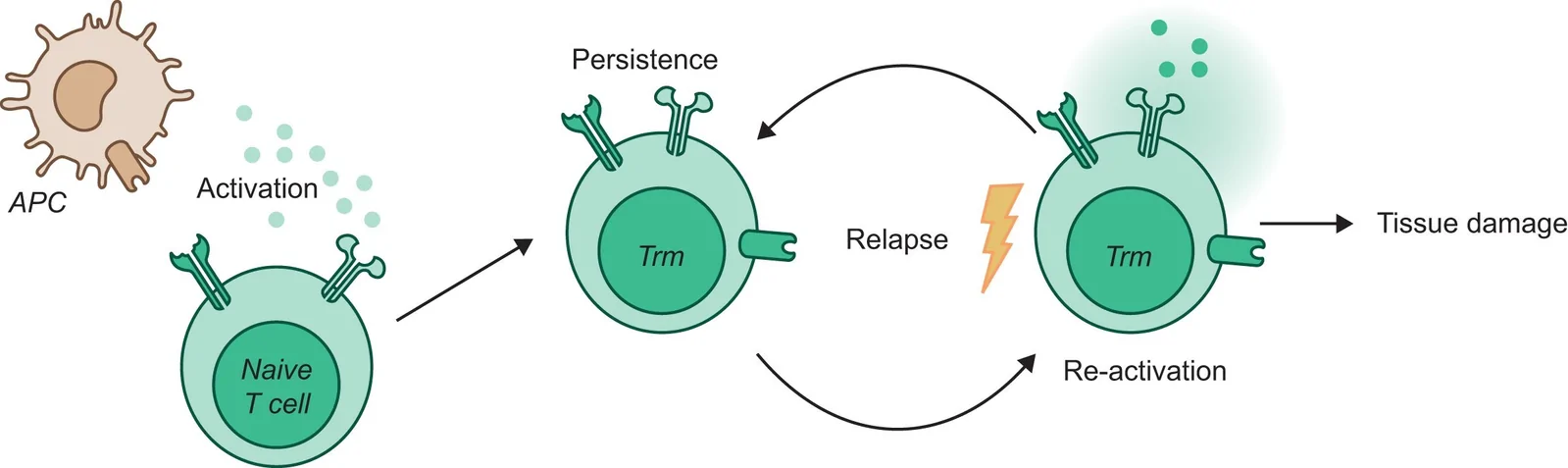
Generation and reactivation of tissue-resident memory T helper cells. Trm cells can arise from activated naive T cells and persist within the tissue. In relapsing disease, Trm cells are reactivated in a TCRindependent manner, promoting a proinflammatory environment and causing tissue damage. After reactivation from inflammatory stimuli, Trm cells can again persist in the tissue site, even after inflammation is resolved.

Long-term persistence of Trm cells within tissues depends on specific metabolic adaptations. Trm cells take up exogenous free fatty acids to persist within the tissues. Inhibition of fatty-acid-binding proteins 4 and 5 (FABP4, FABP5) can impair Trm cell survival [69]. However, currently there are no FABP4/5 inhibitors available for clinical use, although substances have been developed [70].

IL-15 signaling plays an important role in development and maintenance of Trm cells [71]. In preclinical models the long-term inhibition of the IL-2 receptor beta subunit (CD122), which is part of the heterotrimeric IL-15 receptor, resulted in a depletion of Trm cells in the tissue. Involvement of IL-15 signaling is described for the pathophysiology of multiple autoimmune diseases [72–74]. In patients with LN IL-15 promotes cytotoxic T-cell function and might increase tissue damage [75], although a link to tissue resident memory T cells was not shown. Despite these indications for IL-15 inhibition for future targeting of T cells in immune-mediated kidney diseases, the IL-15 signaling pathway was also described as having protective effects on podocytes in the context of focal segmental glomerulosclerosis and in a preclinical model of anti-GBM disease by increasing STAT5 activation [76].

The modulation of S1P signaling could be an additional interesting target, as it might decrease the Trm retention in the tissue [77]. This treatment option is currently mainly used as disease modifying therapy in multiple sclerosis, with the greatest effect coming from inhibition of immune cell egress from the lymph node. Furthermore, the safety profile limits its applicability in immune-mediated kidney diseases.

Regulatory T cells (Treg) physiologically maintain an immunological balance, limiting proinflammatory processes by forkhead box P3 (FOXP3)-dependent production of IL-10. Dysregulation of Treg cells can be observed in LN [78] and ANCA glomerulonephritis [79]. Restoring this dysregulation is a potential therapeutic target.

Advances in cell-based therapies lead to the development of CAR Treg cells. CAR Treg cells are generated from primary Treg cells or by additional transduction of a *FOXP3* gene alongside the CAR [80]. These CAR Treg cells are less dependent on IL-2 stimulation compared with conventional Treg cells and are able to migrate deeply into target tissues.

In preclinical models of SLE, anti-CD19 CAR Treg cells showed promising effects on resolving inflammation [81]. CAR Tregs are currently evaluated in renal transplant rejection and in cases of refractory autoimmune disease (NCT04817774, NCT07473154). Unfortunately, there are currently no trials specifically addressing immune-mediated kidney diseases.

T cell–mediated rejection (TCMR) remains a relevant complication after kidney transplantation associated with worse long-time outcome [82]. The established treatment utilizing broad immunosuppression with steroids and in steroid-resistant cases, anti-thymocyte globulin [83]. Although evidence on T cell–mediated memory response in TCMR is sparse, an increased rate of terminally differentiated effector memory CD8^+^ T cells seemed to be associated with decreased graft survival [84]. The mechanism leading to this worsening in outcome remains unknown. Preclinical models of TCMR indicate a potential involvement of tissue-resident memory T (T_RM_) cells within the inflammatory processes of the kidney [85]. Unfortunately, convincing investigations of the role of human T_RM_ cells in TCMR are lacking.

## CONCLUSION

Disease relapses and thereby mechanisms of immunological memory contribute significantly to the outcome of immune-mediated kidney diseases. Currently, treatment options for relapsing diseases are limited, often requiring individualized treatment concepts. Among the currently best understood mechanisms, B-cell memory contributes substantially to several immune-mediated kidney diseases. Highly differentiated memory B cells or plasma cells resist conventional anti-CD20 treatment and persist in the tissue. Activation of these long-lived B cells can lead to recurrence of disease. To close the therapeutic gap in targeting B cells, CAR-T cells show promising results by migrating deeply in tissue sites and persisting as living therapeutic agents. Additionally, CAAR-T cells could enable highly specific clearance of pathogenic B cells in the future.

Nevertheless, besides B-cell memory, central mechanisms of immune memory in immune-mediated kidney diseases are largely missing. So far, comprehensive data on trained immunity in these diseases is missing. Additionally, epigenetic and metabolic reprogramming in innate as well as adaptive immunity adds complexity to these processes but might enable a variety of novel therapeutic targets in the future. Initial efforts to reverse maladaptive epigenetic programs are already underway and may eventually be applicable to immune-mediated kidney diseases.

Obtaining a better understanding is crucial for the development of specific therapies targeting immune memory. With the new and still improving deep-profiling technologies on one hand and emerging therapeutic advances in cell targeting on the other, we today have the great opportunity to generate long-lasting or even potentially curative treatments for autoimmune diseases including the kidney.

## Data Availability

No new data were generated or analyzed in support of this research.
